# Inferring the Degree of Relatedness and Kinship Types Using an All-in-One Marker Set

**DOI:** 10.3390/genes16040455

**Published:** 2025-04-15

**Authors:** Ran Li, Yu Zang, Jiajun Liu, Enlin Wu, Riga Wu, Hongyu Sun

**Affiliations:** 1 Medical College, Jiaying University, Meizhou 514031, China; liran35@mail.sysu.edu.cn; 2 Faculty of Forensic Medicine, Zhongshan School of Medicine, Sun Yat-sen University, Guangzhou 510080, China; zangy3@mail2.sysu.edu.cn (Y.Z.); liujj258@mail2.sysu.edu.cn (J.L.); wuenlin3@mail2.sysu.edu.cn (E.W.); wurg3@mail.sysu.edu.cn (R.W.); 3 Guangdong Province Translational Forensic Medicine Engineering Technology Research Center, Sun Yat-sen University, Guangzhou 510080, China

**Keywords:** kinship inference, kinship chain, genetic markers, identity by state (IBS)

## Abstract

Background/Objectives: Kinship inference is commonly adopted in various forensic applications, but previous studies have often lacked precision. Methods: In this study, a new method for the nomenclature of kinship types, i.e., kinship chain (KC), was proposed, and then, six types of identity by state (IBS) scores were calculated for simulated and real families using four types of markers. Finally, several Bayesian network (BN)-based classifiers were constructed to investigate the efficiency of the kinship inference. Results: A total of 7, 22, 58, and 3 KCs were obtained for common first-, second-, and third-degree relatives and unrelated pairs, respectively. High accuracies could be achieved in distinguishing between related and unrelated pairs after combining the four types of genetic markers, with an accuracy of >99.99% for all 7 KCs of first-degree relationships and ~99% for 14 out of 22 KCs of second-degree relatives. When comparing relationships of the same degree, the accuracies were 99.28%, 42.31%, and 15.82% for first-, second-, and third-degree relationships, respectively. When it came to differentiating unspecific relationships, the overall accuracy was over 80%. All the results were validated on real family data. Conclusions: With the new nomenclature method of kinship types and the combination of autosomal and non-autosomal genetic markers, kinship inference can be realized with high accuracy and precision, which will be helpful in complex forensic cases, such as the identification of mass disaster victims.

## 1. Introduction

The determination of genetic relatedness is frequently adopted in several forensic applications, such as individual identification of missing persons (MPI) and disaster victims (DVI), inheritance disputes between illegitimate children, and immigration cases [1,2,3]. In most cases, first (1st)- and second (2nd)-degree relationships are involved, including parent–offspring, full siblings, half-siblings, grandparent–grandchild, and avuncular relationships, and less commonly, third (3rd)-degree relationships may be encountered, e.g., first cousins and great-grandparent–great-grandchild.

Conventionally, the relationships are determined using the likelihood ratio (LR) method when LRs exceed a predetermined threshold [4]. Currently, parent–child testing can be reliably performed using marker sets of 13–22 autosomal short tandem repeats (STRs), with additional markers (e.g., single nucleotide polymorphisms (SNPs)) incorporated when mutations are detected to resolve ambiguities [5,6]. However, these marker sets are still insufficient for resolving second- and third-degree relationships, which require substantially more genetic data for robust discrimination [7,8,9,10]. Although previous studies have shown that more markers provide greater power, most of them have mainly focused on autosomal (A) markers, ignoring the value of sex-linked markers, such as markers on the bi-parentally inherited X chromosome (X), paternally inherited Y chromosome (Y), and maternally inherited mitochondrial DNA (mtDNA; M). In theory, fathers and sons should share Y haplotypes and mothers and offspring should share mtDNA haplotypes. Fathers and daughters and mothers and offspring must share at least one allele at each locus on the X chromosome. They are useful in cases of deficiency paternity testing, e.g., in cases with an unusual number of mutations [5,11] or when close relatives of the alleged father are involved [12]. Besides first-degree relationships, these markers can also be useful and can even be used to exclude a relationship for a second-degree relationship, which is not possible with autosomal markers. For example, a pair of paternal half-sisters must share a common X haplotype inherited from their father, and the claimed relationship can be rejected if they do not share any alleles at any X-chromosomal marker (ignoring mutations). If mutations are taken into account, the efficiency can also be significantly increased by adding just a few X-chromosomal markers [8]. Therefore, there is a potential improvement in accuracy by combining multi-types of markers for forensic kinship analysis.

**Figure 1:**
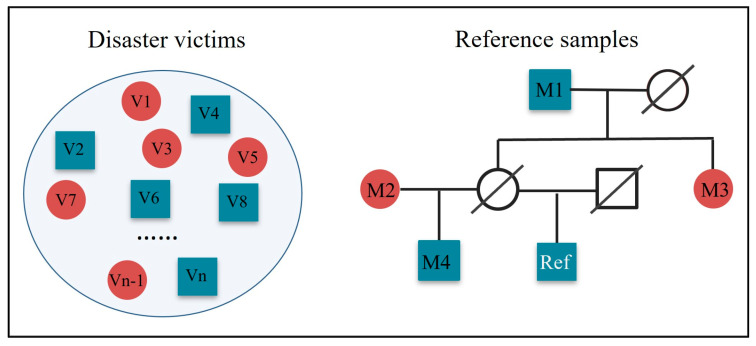
An example case of disaster victim identification. V: victim; M: missing person; Ref: reference samples. Red circles represent females and green squares represent males.

On the other hand, conventional forensic kinship testing compares the likelihood that two individuals are related as a specified relationship against that they are unrelated. In some circumstances, DNA is available from two individuals but the relationship between them is uncertain, which is common in disaster victim identifications, especially in cases involving multiple victims, often referred to as mass identifications [13,14,15]. Pairwise blind searching is often performed to identify familial relationships with the victims, a necessary step to infer the genetic and familial composition of the victim samples. This approach is also useful for identifying familial relationships between the victims and reference samples, which can mitigate issues arising from unexpected pedigree relationships [14]. Challenges emerge in some cases, and an example of such a case is shown in Figure 1. In this example, we may succeed in finding that the reference sample (Ref) is related to the three missing persons (M), i.e., M1, M3, and M4, if a large number of independent autosomal markers are detected. However, it will be difficult to conclude how exactly they are related, as they all belong to second-degree relationships and have identical LR distributions. In fact, the addition of non-autosomal markers can be used to address this problem. Among the three relatives of Ref (i.e., M1, M3, and M4), M3 can be easily identified by her sex. The differentiation between M1 and M4 can also be achieved by adding mtDNA markers as M4 and Ref share the same mtDNA haplotype, whereas M1 and Ref do not. Given this, a high resolution of kinship analysis can be expected by combining autosomal and non-autosomal markers.

In this study, we first introduced a new method for the nomenclature of kinship types, i.e., the kinship chain (KC), which links the sex combinations of two individuals of interest and all related family members. Then, six types of identity by state (IBS) scores were calculated for simulated and real families using markers on the autosome, X-chromosome, Y-chromosome, and mtDNA. Finally, we constructed several Bayesian network (BN) models to explore the efficiency of distinguishing between related and unrelated individuals as well as between different KCs.

## 2. Materials and Methods

### 2.1. Samples

In this study, 108 unrelated individuals (52 males and 56 females) from a Han Chinese population, 69 members from a big family (Supplementary Figure S1a), and 8 individuals from three small families (Supplementary Figure S1b) were recruited with informed consent. Two milliliters of peripheral blood was collected from each individual. DNA was extracted using QIAamp DNA Blood Mini Kits (Qiagen, Hilden, Germany) and quantified using Qubit dsDNA BR Assay Kit (Thermo Fisher Scientific, San Francisco, CA, USA) on a Qubit 3.0 fluorometer. This study was approved by the Ethics Committee of Sun Yat-sen University (Guangzhou, China), with an approval number of [2019]064.

### 2.2. Library Preparation and Sequencing

DNA libraries were constructed using the MGIEasy Signature Identification Library Prep Kit (MGI Tech, Shenzhen, China), with which Amelogenin, 52 A-STRs, 27 X-STRs, 48 Y-STRs, 145 identity informative SNPs (iiSNPs), 53 ancestry informative SNPs (aiSNPs), 29 phenotype informative SNPs (piSNPs), and the hypervariable region of mtDNA were co-amplified [16]. The libraries were then pooled and subjected to the rolling circle amplification (RCA) procedure to generate a mass of DNBs. Finally, these libraries were sequenced using an MGISEQ-2000RS sequencer (MGI Tech, Shenzhen, China). Two independent sequencing runs were conducted in this study: one for the family samples and another for the 108 unrelated individuals.

### 2.3. Data Analysis

#### 2.3.1. Genotype Calling

SOAPnuke [17] was adopted for sequence quality control and clean sequences were aligned to the GRCh37 reference genome using minimap2 [18]. Base calls at these targeted SNPs were counted individually using Bam-readcount [19]. STRait Razor 3.0 [20] was used to extract target sequences for STRs and mtDNA haplotypes from the FASTQ files. To genotype SNP and mtDNA haplotypes, we required a minimum depth of 100× and a threshold of allele coverage ratio (t_ACR_) of 0.1; for STR genotyping, we required a minimum locus-specific depth of 100× and a t_ACR_ of 0.4 (except DYS612; t_ACR_ = 0.45).

#### 2.3.2. Allele Frequencies, Haplotype Frequencies, and Forensic Parameters

Allele frequencies and related forensic parameters were calculated according to [21,22,23] based on the genetic data of the 108 unrelated individuals. The R package *Pegas* [24] was used for Hardy–Weinberg equilibrium (HWE) testing and linkage disequilibrium (LD) testing for autosomal and X-chromosomal markers. For Y-STR and mtDNA, haplotype diversity (HD) was calculated as HD = [N (1 − ∑p_i_^2^)]/(N − 1), where N was the number of haplotypes and p_i_ was the frequency of the ith haplotype.

#### 2.3.3. Pedigree Simulation

Assuming HWE and linkage equilibrium (LE), five thousand pedigrees (Supplementary Figure S2) were simulated using an in-house R script. For autosomal markers, the alleles of founders were randomly assigned according to the allele frequencies of each locus. Founders transmitted a single allele to his/her offspring with equal probability. Mutations were also incorporated, with a rate of 0.002 for STRs and 1 × 10^−8^ for SNPs. Genetic positions of related markers were linearly interpolated using the sex-averaged genetic map established by Bherer et al. [25], and the recombination rates (Rc) between two markers were estimated using the Kosambi function [26]. Genotypes on the same chromosomes were rearranged based on Rc before transmission to a child.

For X-chromosomal markers, the genotypes were generated in the same way as that of autosomal markers for females, while males transmitted an X-STR haplotype to their daughters with a mutation rate of 0.002 at each locus. Y-STR haplotypes were randomly selected from Section 2.3.2 and assigned to male founders of each pedigree. Males transmitted a Y-STR haplotype to their sons with a mutation rate of 0.002 at each Y-STR. MtDNA haplotypes were randomly selected from Section 2.3.2 and assigned to the founders of each pedigree. Females transmitted corresponding mtDNA haplotypes to their offspring without mutation and heterogeneity.

#### 2.3.4. Kinship Nomenclature

**Figure 2:**
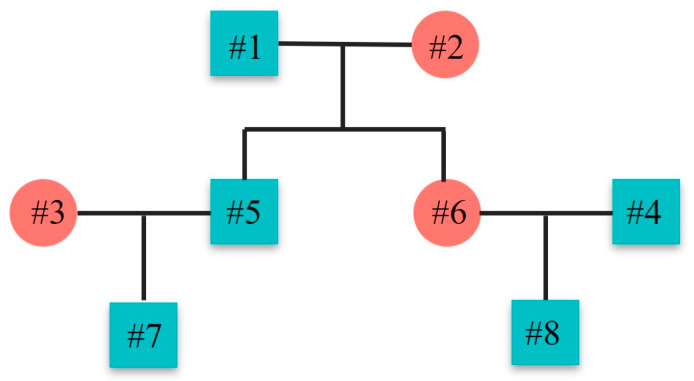
An example of simulated pedigree. Red circles represent females and green squares represent males.

We defined a new method of kinship nomenclature, i.e., kinship chain (KC), which is a continuous string and consists of arrows and the sex combinations of two individuals and their mutual relatives. For example, #1 and #8 (Figure 2) are grandparent–grandchild relationships and they are connected by #6. The sexes of #1, #6, and #8 are male (M), female (F), and male, respectively. The KC between #1 and #8 is then expressed as “M→F→M”. Similarly, #7 is a first cousin to #8 and they are connected by #1, #2, #5, and #6. Of the six family members, #1 and #2 are couples and expressed as “←FM→”. The KC between #7 and #8 can be expressed as “M←F←FM→M→M” (or “M←M←FM→F→M”). For paternal half-sisters, the KC is “F←M→F”, where “←M→” means that the two females share a common male ancestor. For unrelated male–male, female–male and female–female pairs, the KCs are “MM”, “FM”, and “FF”, respectively. In total, 7, 22, 58, and 3 KCs were obtained for common 1st-, 2nd-, and 3rd-degree relatives and unrelated individual pairs, respectively, resulting in a total of 90 KCs for the four main kinship categories (Supplementary Table S1).

#### 2.3.5. IBS Scores

Several types of IBS scores were calculated for each KC, i.e., ibs, *IBS*, *ibs0*, and *IBS0*. We defined *ibs* as the number of shared alleles at a locus between two individuals, which can be 0, 1, and 2. Correspondingly, *IBS* denotes the sum of *ibs* across multiple markers. We assigned *ibs0* = 1 if no alleles were shared at a locus; otherwise, *ibs0* = 0. *IBS0* denotes the sum of *ibs0* across multiple markers. The calculation of *ibs* and *ibs0* for autosomal, X-chromosomal, Y-chromosomal, and mtDNA markers are summarized in Supplementary Table S2. Notably, we defined *ibs* = 1 if two individuals share an identical mtDNA haplotype; otherwise, *ibs* = 0. As two types of autosomal markers (A-STR and A-SNP) were included, we normalized IBS scores as

$$A-IBS=\frac{{A-IBS}_{STR}}{N_{STR}\times 2}+\frac{A{-IBS}_{SNP}}{N_{SNP}\times 2}$$

$$A-IBS0=\frac{{A-IBS0}_{STR}}{N_{STR}}+\frac{{A-IBS0}_{SNP}}{N_{SNP}}$$

where *N_STR_* and *N_SNP_* represent the numbers of *A-STR* and *A-SNP*, respectively. In total, six types of *IBS* scores were calculated, including *A-IBS*, *A-IBS0*, *X-IBS*, *X-IBS0*, *Y-IBS*, and *M-IBS*.

#### 2.3.6. Kinship Inference

Five thousand pedigrees were simulated and IBS scores (*A-IBS*, *A-IBS0*, *X-IBS*, *X-IBS0*, *Y-IBS*, and *M-IBS*) were calculated for each KC according to Section 2.3.5. We then discretized these IBS scores using the R package *infotheo*. Several Bayesian networks (BNs) were constructed to classify pairwise relationships, and the performance was evaluated using 5-fold cross-validation. Finally, these models were validated using real family data. All the simulations and calculations in this study were performed using *R* version 3.6.1 [27].

## 3. Results

### 3.1. Forensic Parameters, HWE Testing and LD Testing

On average, 2.12 million reads (ranging from 802,303 to 4,361,875) were obtained for each sample and read counts at each marker are summarized in Supplementary Table S3. The average number of alleles observed per locus varied between marker types: 8.83 for A-STRs, 7.78 for X-STRs, 5.85 for Y-STRs, 2 for iiSNPs, 1.83 for aiSNPs, and 1.45 for piSNPs. Of note, 9 aiSNPs and 25 piSNPs were found to be monomorphic in the studied population. Twelve iiSNPs (i.e, rs1343469, rs1355634, rs1512612, rs1657695, rs1657741, rs1698647, rs2356027, rs3094868, rs3817211, rs62431284, rs6499422, and rs929310) showed significant deviations from HWE after Bonferroni correction (*p* < 0.05/145) due to homologous sequences on the genome and nonspecific amplification during PCR. Similar results have also been previously reported [28], and therefore, the twelve iiSNPs were excluded from subsequent analyses. LD testing was then performed for the 52 A-STRs and 133 iiSNPs. The results showed that 15 pairs were in LD after Bonferroni correction (*p* < 0.05/17,020). Among these, 14 pairs were located on different chromosomes, likely reflecting random associations. Only one pair (rs2235907-rs8124995) was located in close physical proximity on the same chromosomes and was considered to represent genuine LD. For X-chromosomal markers, six significant LD pairs were identified in males (with inter-marker distances ranging from 16.44 to 51.65 Mb) and two pairs in females (35.76 and 71.69 Mb). Given the substantial physical distances between these markers (>16 Mb) and the lack of concordance between male and female results, these associations were attributed to random effects rather than true LD. Consequently, all X-chromosomal markers were considered to be in LE.

After excluding the twelve iiSNPs out of HWE and one of the two iiSNPs in LD, the combined match probability, the combined power of exclusion (CPE) for duo paternity testing (CPE_duo_), and the CPE for trio paternity testing (CPE_trio_) were 4.93 × 10^−110^, 1–2.00 × 10^−19^, and 1–4.99 × 10^−32^, respectively, using the remaining 52 A-STRs and 132 iiSNPs. For X-STRs, the power of discrimination (PD) in males, PD in females, the mean exclusion chance (MEC) in father–daughter duos, and the MEC in trios with daughters were 1–2.99 × 10^−16^, 1–1.40 × 10^−26^, 1–2.16 × 10^−10^, and 1–1.12 × 10^−14^, respectively. For Y-STRs, all the haplotypes were unique due to the large set of Y-STRs despite the small sample size of this study. In total, 106 mtDNA haplotypes were observed in the 108 individuals, 2 of which were observed twice (i.e., “73G, 207A, 248A-del, 263G, 302.1C, 310.1C, 16093C, 16114A, 16260T, 16298C, 16355T, 16362C” and “73G, 248A-del, 263G, 302.1C, 310.1C, 514C-del, 16108T, 16129A, 16162G, 16172C, 16214T, 16304C”), resulting in a haplotype diversity of 0.9996539. Due to the low polymorphisms of aiSNP and piSNP, they were not included in the following analyses.

### 3.2. IBS Score Distributions

**Figure 3:**
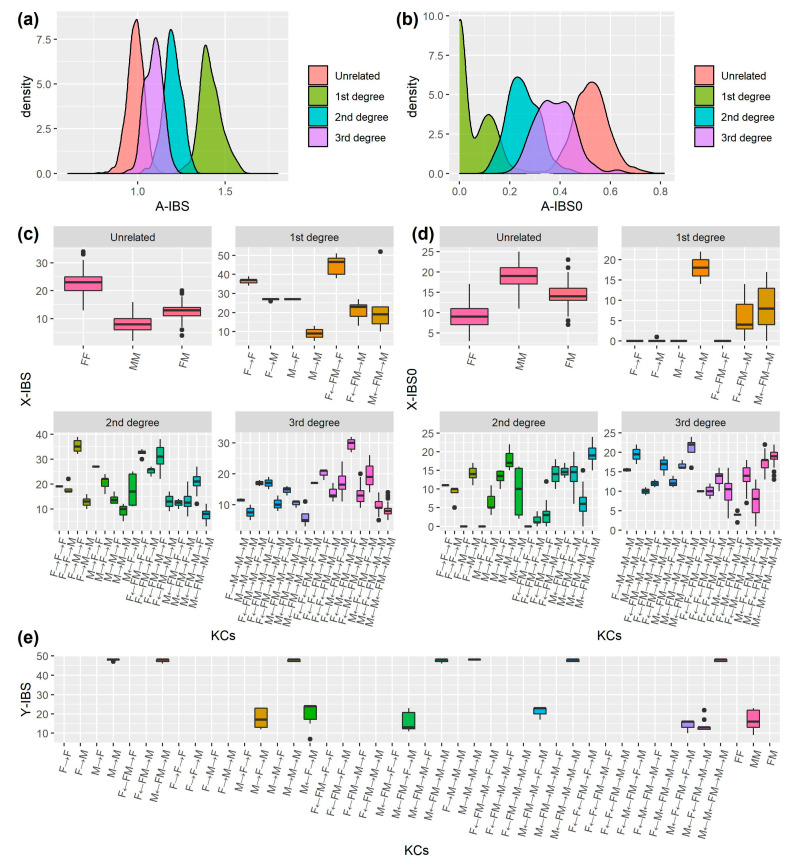
The IBS score distributions of A-IBS (**a**), A-IBS0 (**b**), X-IBS (**c**), X-IBS0 (**d**), and Y-IBS (**e**) for different kinship chains (KCs) based on genetic data of the four real families. A total of 496 pairs of related and 1693 pairs of unrelated individuals were included; see Supplementary Table S4 for details.

In this study, we focused exclusively on first- to third-degree relationships and unrelated individuals (UN). Based on the four studied families, 44 out of 90 KCs were obtained and the number of these KCs ranged from 1 to 41 pairs for relatives and from 309 to 904 for UN pairs, resulting in a total of 496 pairs of relatives and 1693 pairs of unrelated individuals (Supplementary Table S4). We further calculated the IBS scores using the genotypes of 52 A-STRs, 27 X-STRs, 48 Y-STRs, 132 iiSNPs, and mtDNA haplotypes. The distributions of *A-IBS*, *A-IBS0*, *X-IBS*, *X-IBS0*, and *Y-IBS* for different KCs are shown in Figure 3. On the whole, *A-IBS* decreased consistently for first-, second-, and third-degree relatives and UN pairs (Figure 3a). The mean and standard deviation of *A-IBS* scores for the four main kinship categories were 1.42 ± 0.06, 1.20 ± 0.05, 1.09 ± 0.05, 0.99 ± 0.05, respectively. The relatively large overlap between first- and third-degree relationships can be explained by their high level of shared DNA segments. Distribution in reverse order was observed for *A-IBS0* and a double peak was observed for the first-degree relatives (Figure 3b), which corresponds to parent–offspring relationships and full siblings, respectively.

When it comes to *X-IBS*, different KCs had different *X-IBS* distributions even though they were from the same degrees of relatedness. *X-IBS* was associated not only with the degree of relatedness but also with the sexes of the two individuals and their mutual relatives. Full sisters (“F←FM→F”) had the highest averaged *X-IBS* (45.33 ± 4.60) among the first-degree relationships, followed by mother–daughter pairs (“F→F”; *X-IBS* = 36.47 ± 1.36). For the second- and third-degree relatives, the highest averaged *X-IBS* were observed at the paternal grandmother–granddaughter pairs (“F→M→F”; *X-IBS* = 35.33 ± 2.80) and the female cousins (“F←M←FM→M→F”; *X-IBS* = 29.60 ± 2.07), respectively. Not surprisingly, several KCs (e.g., “M→M”, “M→M→M”, “M←FM→M→M”, “M→M→M→M”, “M←M←FM→M→M”, “M←FM→M→M→M”) showed similar IBS distributions to their sex-match UN pairs (Figure 3c) as they shared no segment identity by decent on X chromosome. Some KCs showed zero or near zero *X-IBS0*, including “F→F”, “F→M”, “M→F”, “F←FM→F”, “F→M→F” and “F←FM→F→F” (Figure 3d).

With respect to *Y-IBS* and *M-IBS*, all the male relatives from the same paternal lineages showed much higher IBS scores (47.65 ± 0.51) than those from different paternal lineages (16.04 ± 4.89) and UN male pairs (17.32 ± 4.37; Figure 3e). Similarly, all the relatives from the same maternal lineages had identical mtDNA haplotypes, while they were all different for those from different maternal lineages and UN pairs. It is worth mentioning that different haplotypes were initially identified among B1, B3, and B6 (as full siblings of each other) as a result of mtDNA heteroplasmy at nt 310. The major allele was 310.1C and the minor allele was 310.2C for both B1 and B3 while reverse allele composition was observed at B6. For details of IBS scores (*A-IBS*, *A-IBS0*, *X-IBS*, *X-IBS0*, *Y-IBS*, and *M-IBS*), please refer to Supplementary Table S4.

### 3.3. Distinguish Between Relatives and Unrelated Pairs

Considering the small sample size and the lack of some common relationships with the families studied (Supplementary Figure S1 and Table S4), we used simulated family data (Supplementary Figure S2; *n* = 5000) to further estimate the efficiency of distinguishing between 87 related KCs and their sex-matched unrelated KCs. First, the dependence among the six IBS scores (A-IBS, A-IBS0, X-IBS, X-IBS0, Y-IBS, and M-IBS) was explored. We found that there was a significant association between (i) A-IBS and A-IBS0, and (ii) X-IBS and X-IBS0, while no dependence was observed for IBS scores of different types of markers for many relationships. However, the association may vary for different relationships (Supplementary Figure S3). Given this, we constructed a classifier for each group (each consisting of a related KC and a sex-matched unrelated KC) using the Bayesian network model, which provides a probabilistic and graphical framework for modeling high-dimensional joint distributions with complex correlation structures. An example of BN topology structure is shown in Supplementary Figure S4.

**Figure 4:**
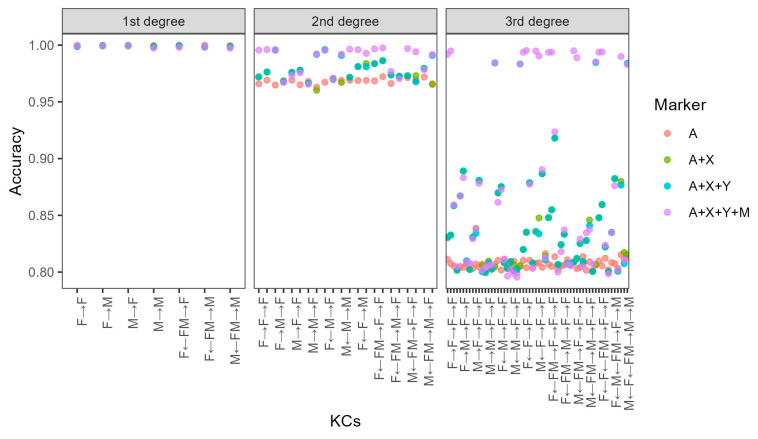
Accuracies for the differentiation between 87 related KCs and their sex-matched unrelated pairs when using autosomal (A), X-chromosomal (X), Y-chromosomal (Y), and mtDNA (M).

With simulated data and five-fold cross-validation, the averaged accuracies were 99.99%, 97.43%, and 83.07% for first-, second-, and third-degree relatives, respectively. These values increased significantly with the addition of X-chromosomal, Y-chromosomal, and mtDNA markers (Figure 4). After adding X-STRs, the highest increase was observed at paternal grandmother–granddaughter (“F→M→F”) and paternal half-sisters (“F←M→F”; Figure 4 and Supplementary Figure S5). This was expected because both relationships must share one IBD allele across the entire X chromosome. When the four types of genetic markers were combined, accuracies increased to about 99% for 14 out of 22 KCs of second-degree relatives. For third-degree relatives, accuracy rates were all about 99% for male KCs of the same paternal lineages after adding Y-STRs and for KCs of the same maternal lineages after adding mtDNA.

These models were further validated with real family data. All of the 145 pairs of first-degree relatives were correctly assigned and only 1 pair of paternal grandfather–granddaughter (“M→M→F”) was misclassified as unrelated individuals for second-degree relatives, thus resulting in an overall accuracy of 100% and 99.53% for first- and second-degree relatives, respectively. For third-degree relatives, 123 out of 138 (89.13%) pairs were correctly assigned, of which KCs of the same paternal or maternal lineages were all correctly assigned.

### 3.4. Distinguish Relationships of the Same Degree of Relatedness

**Figure 5:**
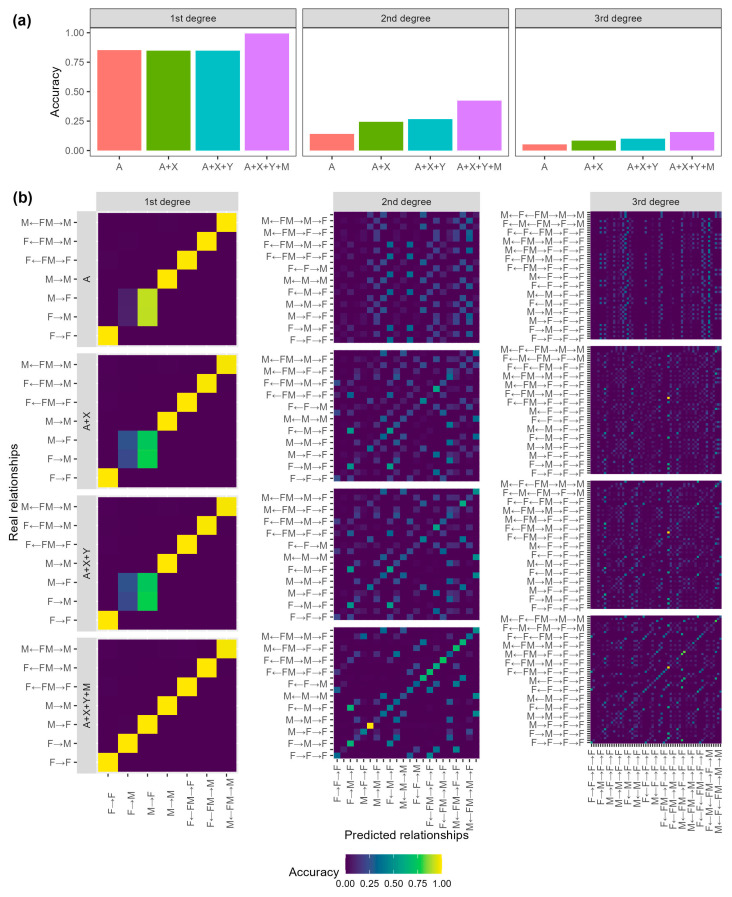
Overall accuracies (**a**) and detail results (**b**) for the differentiation of KCs within first-, second-, and third-degree relationships when using markers on the autosome (A), X-chromosome (X), Y-chromosome (Y), and mtDNA (M).

We also explored the efficiency of distinguishing relationships of the same degree of relatedness based on the simulated dataset as described above. On the whole, accuracy decreased constantly for more distant relationships when using the same marker sets (Figure 5a). If merely autosomal markers were included, 85.17% of first-degree relationships were correctly assigned. In contrast, the rate was only 14.05% for second-degree relationships and 5.17% for third-degree relationships, respectively, which were very close to a random probability, 13.64% (3/22) for second-degree relationships and 5.17% (3/58) for third-degree relationships. Accuracy increased significantly after the addition of non-autosomal markers and the highest increase was observed when including *M-IBS* for all the three main categories. When four types of markers were combined, the accuracy was 99.28%, 42.31%, and 15.82% for first-, second-, and third-degree relationships, respectively (Figure 5a). These models were further validated with real family data. The accuracies were 99.31% (144/145), 44.60% (95/213), and 14.49% (20/138) for first, second, and third-degree relationships, respectively, which were consistent with those based on simulated data.

For the first-degree relationships, *A-IBS0* made great contributions to the differentiation between parent–child and full siblings. However, it cannot be used to distinguish the four parent–child KCs. Most misclassifications of this main category were observed between father–daughter (“M→F”) and mother–son (“F→M”) pairs when using autosomal, X-chromosomal, and Y-chromosomal markers (Figure 5b). The two KCs had very similar inheritance patterns at autosome and X-chromosome and *Y-IBS* was of no use due to different sex. The differentiation was finally achieved by *M-IBS* as “F→M” shared the same mtDNA haplotypes while “M→F” did not. Of the 22 KCs of second-degree relationships, most KCs had accuracies lower than 70% even though four types of markers were combined. However, the rate for maternal grandfather–grandson (“M→F→M”) was much higher (90.63%), which may be explained by its distinct inheritance patterns. For the third-degree relationships, accuracies differed greatly among different KCs, with the highest at “F←FM→F→M→F” (56.44%) and the lowest at “M→M→M→F” (9.03%).

### 3.5. Estimate an Unspecific Relationship

**Figure 6:**
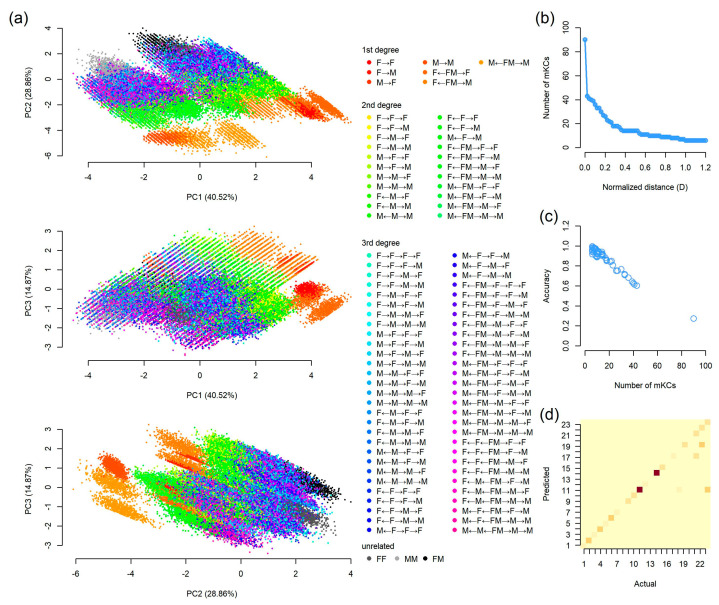
Results for distinguishing 90 KCs based on the six IBS scores (A-IBS, A-IBS0, X-IBS, X-IBS0, Y-IBS, and M-IBS). (**a**) Scatter plots of principal component analysis (PCA); (**b**) the relationship between normalized distance and the number of mKCs; (**c**) the relationship between the number of mKCs and accuracy; and (**d**) a heatmap of actual and predicted results based on real family data with normalized distance (D) = 0.22.

Finally, we explored the efficiency of estimating an unspecific relationship based on the simulated dataset as described above. The 90 KCs were visualized using principal component analysis (PCA) based on the six IBS scores (*A-IBS*, *A-IBS0*, *X-IBS*, *X-IBS0*, *Y-IBS*, and *M-IBS*). The results showed that the first three components explained 40.37%, 28.90%, and 14.92% of the total variance, respectively, thus resulting in a cumulative proportion of nearly 85% (Figure 6a). The seven KCs of first-degree relationships were positioned separately from each other as well as with KCs of second- and third-degree relationships and UN pairs. However, there were considerable overlapping areas within and among some KCs of second- and third-degree relationships, indicating potential difficulty in differentiating these KCs. Given this, we merged some of these KCs, which are referred to as merged KC (mKC) hereafter. Briefly, we calculated the centroid for each KC and defined KCs with normalized distance (D) less than pre-defined thresholds as one mKC. Then, a BN-based classifier was constructed and evaluated using five-fold cross-validation.

The results show that the overall accuracy was only 27.31% when 90 KCs were independently assigned as a single mKC. If KCs with D < 0.02 were merged, 43 mKCs were obtained and the accuracy increased significantly to 60.28%. The numbers of mKCs decreased quickly with increased Ds (Figure 6b) and was linearly corelated with accuracy (Figure 6c). We found that if D = 0.22, 23 mKCs could be generated and the accuracy was over 80%. Of the 23 mKCs, the seven KCs of first-degree relationships and the three KCs of UN pairs were individually assigned as a single mKC (Supplementary Table S5). Among the 22 KCs of second-degree relationships, only maternal grandfather–grandson (“M→F→M”) was assigned as a single mKC, which was consistent with the results above (Section 3.4).

Then, the BN classifier, which was constructed based on simulated family data and the 23 mKC nomenclature system, was validated using real family data. Considering the much larger numbers of UN pairs (*n* = 1693) than sex-matched relatives (*n* = 496; Supplementary Table S4), we randomly selected 50 pairs of mKCs 21–23 from the 1693 unrelated pairs, thus resulting in a total of 646 real pairs. As shown in Figure 6d, except for two pairs, all the seven mKCs (mKCs 1–7) corresponding to first-degree relationships, were correctly assigned, while 32 out of 351 (9.12%) pairs of mKCs 8–20 were misclassified. We found that most misclassifications (83/117) were observed at mKCs belonging to unrelated relationships, i.e., mKCs 21–23, indicating a slight bias to false positive predictions of the classifier. The overall accuracy was 81.89% (529/646), which was very close to that based on simulated data (80.52%).

## 4. Discussion

This study introduced a new method of kinship nomenclature, i.e., kinship chain (KC), which may promote overcoming the ambiguity in naming close relationships and difficulty in naming complex or distant relationships. This is also the basis for a high resolution of kinship analysis. Another contribution of this study is that we demonstrated the feasibility and efficiency of distinguishing between related and unrelated individuals as well as among different relationships of the same and/or different degrees of relatedness, by combining multi-types of forensic genetic markers (autosomal, X-chromosomal, Y-chromosomal, and mtDNA) and the Bayesian network.

The numbers and types of genetic markers differ in different cases for kinship analyses [7,12,29,30,31]. However, with the traditional capillary electrophoresis (CE)-based genotyping method, analyzers have to detect these markers separately, which is time-consuming and laborious. In addition, due to the limitation in the number of tested markers, it is generally not sufficient enough to perform a distant kinship analysis. Reference samples of first-degree relationships (parent–offspring and full siblings) are preferred for missing person identification and familial searching [1,32]. Fortunately, with advances in massively parallel sequencing (MPS), we can now detect a large number of genetic markers of the same and (or) different types in a single reaction [6,16,33,34], making it possible to realize precision kinship analysis of common relationships. Benefiting from this, the number and degree of reference samples required for DVI or MPI may change.

Our study showed that, with the new nomenclature method of kinship types and the combination of autosomal and non-autosomal genetic markers, a higher resolution of kinship analysis can be realized with high accuracy. If the four types of genetic markers were combined, the overall accuracies for the differentiation between related and unrelated pairs, were 100% of all KCs of first-degree relationships, about 99% of 14 out of 22 KCs of second-degree relationships, and 99% for KCs from the same maternal and/or paternal lineages of third-degree relationships, indicating that relatives of these kinds could be chosen as good reference samples. In order to reconstruct the pedigrees within victims and validate the relationships among reference samples, it would be helpful to infer the relationships between two individuals with high resolution and confidence. However, it is a challenge to differentiate relationships of the same degree of relatedness, which share identical LR distributions and cannot be identified using conventional autosomal markers. Nevertheless, our study showed that combining multi-types of genetic markers may be a solution. The accuracies were 99.28%, 42.31%, and 15.82% for first-, second-, and third-degree relationships, respectively (Figure 5). In particular, the seven KCs of first-degree relationships and “M→F→M” (maternal grandfather–grandson) of second-degree relationships showed distinct inheritance patterns and could be identified with very high accuracy. Although it was not possible to differentiate all these 90 KCs (27.31% in accuracy), we could improve the performance by merging some KCs. Despite the reduction in resolution, accuracy improved significantly (Figure 6c,d). The model was further validated using real family data and the overall accuracy was 81.89%, consistent with that based on simulated data. It is worth noting that the real mKCs of the 86 out of 117 (73.50%) misclassified pairs ranked as the second highest probabilities. If the top two mKCs were considered correct classifications, the accuracy was much higher (95.20%).

Furthermore, previous studies, using likelihood-based methods, have also shown that linked markers can be employed to distinguish relationships of the same degree of relatedness, despite a potential computational burden in large datasets [35,36]. Morimoto et al. [37] compared the difference in chromosomal sharing segments and succeeded in differentiating collateral relationships from lineal relationships of the same degree of kinship. Therefore, a higher resolution or accuracy of kinship inference can be expected with the combination of these methods.

The pairwise blind search is a critical step in DVI and MPI, serving three key purposes: (1) inferring the genetic and familial composition of victim samples, (2) validating relationships among reference samples, and (3) detecting unexpected pedigree relationships [14,32,38]. At this stage, the IBS approach, a model-free method, is generally employed. Notably, this method is also one of the officially recommended methods for the identification of biological full-sibling relationships [39]. Despite it being a relatively lower power compared to the likelihood-based method (Supplementary Figure S6 and in Cui et al.’s study [40]), the IBS approach is computationally simple and much faster, making it particularly suitable for mass disaster scenarios requiring rapid analysis. These advantages position our method as a potential solution for high-throughput pairwise searches in large-scale identification efforts.

There are also some limitations in this study. First, our KC method will be a good alternative to describe a pedigree in text, when a picture is not possible. However, it is not applicable to non-pairwise relationships. In addition, the nomenclature may have difficulties in naming relationships that are in-between two degrees of relationships, e.g., a three-quarter sibling (3/4S) relationship, which shares fewer alleles than a first-degree relationship but more alleles than a second-degree relationship [41]. Second, the MGIEasy identification system is insufficient to distinguish relationships of more than a third degree of relatedness from unrelated pairs. Systems with higher power, such as microarray [42] and whole genome sequencing (WGS) [43], are encouraged. Third, marker dropouts may occur in degraded DNA samples and there may be a reduction in power, particularly if a large proportion of markers are lost.

## 5. Conclusions

With the new nomenclature method of kinship types and the combination of autosomal and non-autosomal genetic markers, kinship inference can be realized with high accuracy and precision, which will be helpful in complex forensic cases, such as the identification of mass disaster victims.

## Data Availability

The original contributions presented in the study are included in the article/Supplementary Material; further inquiries can be directed to the corresponding authors.

## Supplementary Materials

The following supporting information can be downloaded at https://www.mdpi.com/article/10.3390/genes16040455/s1, Supplementary Figure S1: Pedigrees of the four families studied. Solid squares and circles indicate samples that were collected. With the four families, 44 out of 90 KCs can be obtained, resulting in a total of 496 pairs of related and 1693 unrelated individuals; Supplementary Figure S2: Pedigrees of the simulated family; Supplementary Figure S3: Scatter plot of A-IBS, A-IBS0, X-IBS, X-IBS0, Y-IBS, and M-IBS for father–son and unrelated pairs. The father–son relationship (“M→M”) is shown in red points and cyan points indicate unrelated pairs (“MM”). In this example, *X-IBS* correlated perfectly with *X-IBS0* for both “MM” and “M→M”, as the equation of *X-IBS* + *X-IBS0* =27 always holds for any male pair. In addition, there was a significant association between A-IBS and A-IBS0 for “MM” but not for “M→M”; Supplementary Figure S4: An example of Bayesian network topology structure. An arrow indicates an association between the two IBS metrics; Supplementary Figure S5: Accuracies for the differentiation between 87 related KCs and their sex-match unrelated pairs when using autosomal (A), X-chromosomal (X), Y-chromosomal (Y), and mtDNA (M); Supplementary Figure S6: Comparison of the accuracy for kinship inference using LR and IBS methods. This figure is generated based on the genetic data of the four real families. Only autosomal markers, including A-STRs and iiSNPs, were included. If a pair had an LR value greater than 1, they were considered related; otherwise they were unrelated; Supplementary Table S1: Ninety kinship chains (KCs) and corresponding sex combinations, numbers of miosis, degrees of relatedness, relationship types, and sub-relationship types; Supplementary Table S2: Calculation of *ibs* and *ibs0* for autosomal, X-chromosomal, Y-chromosomal, and mtDNA; Supplementary Table S3: Read counts for STR, SNP, and mtDNA; Supplementary Table S4: The number of pairs and IBS scores for 90 KCs based on the four families studied. A-IBS and A-IBS0 were the sums of normalized IBS scores of A-STRs and iiSNPs; Supplementary Table S5: Details of the 23 mKCs and their corresponding KCs.

## References

[1] Ge J., Budowle B., Chakraborty R. (2011). Choosing Relatives for DNA Identification of Missing Persons. J. Forensic Sci..

[2] Kling D., Tillmar A.O., Egeland T. (2014). Familias 3—Extensions and new functionality. Forensic Sci. Int. Genet..

[3] Karlsson A.O., Holmlund G., Egeland T., Mostad P. (2007). DNA-testing for immigration cases: The risk of erroneous conclusions. Forensic Sci. Int..

[4] Annual Report Summary for Testing in 2010. AABB. https://www.aabb.org/docs/default-source/default-document-library/accreditation/rtannrpt10.pdf?sfvrsn=6aabed41_0.

[5] Sun H.Y., Li H.X., Zeng X.P., Ren Z., Chen W.J. (2012). A paternity case with mutations at three CODIS core STR loci. Forensic Sci. Int. Genet..

[6] Wu J.Z., Wang L.X., Yang X.Y., Pan D.H., Lu X.Y., Liu C.H., Han X.L., Liu H., Shi M.S., Liu C. (2022). Forensic application of a novel MPS-based panel (90 STRs and 100 SNPs) in a non-exclusion parentage case with three autosomal STRs incompatibilities. Leg. Med..

[7] Tamura T., Osawa M., Ochiai E., Suzuki T., Nakamura T. (2015). Evaluation of advanced multiplex short tandem repeat systems in pairwise kinship analysis. Leg. Med..

[8] Li R., Li H., Peng D., Hao B., Wang Z., Huang E., Wu R., Sun H. (2019). Improved pairwise kinship analysis using massively parallel sequencing. Forensic Sci. Int. Genet..

[9] Tao R., Xu Q., Wang S., Xia R., Yang Q., Chen A., Qu Y., Lv Y., Zhang S., Li C. (2022). Pairwise kinship analysis of 17 pedigrees using massively parallel sequencing. Forensic Sci. Int. Genet..

[10] Zhang Q., Zhou Z., Wang L., Quan C., Liu Q., Tang Z., Liu L., Liu Y., Wang S. (2020). Pairwise kinship testing with a combination of STR and SNP loci. Forensic Sci. Int. Genet..

[11] Junge A., Brinkmann B., Fimmers R., Madea B. (2006). Mutations or exclusion: An unusual case in paternity testing. Int. J. Legal Med..

[12] Garcia F.M., Bessa B.G.O., Santos E.V.W.D., Pereira J.D.P., Alves L.N.R., Vianna L.A., Casotti M.C., Trabach R.S.R., Stange V.S., Meira D.D. (2022). Forensic Applications of Markers Present on the X Chromosome. Genes.

[13] Skare Ø., Sheehan N., Egeland T. (2009). Identification of distant family relationships. Bioinformatics.

[14] Bertoglio B., Grignani P., Di Simone P., Polizzi N., De Angelis D., Cattaneo C., Iadicicco A., Fattorini P., Presciuttini S., Previderè C. (2020). Disaster victim identification by kinship analysis: The Lampedusa October 3rd, 2013 shipwreck. Forensic Sci. Int. Genet..

[15] Vigeland M.D., Egeland T. (2021). Joint DNA-based disaster victim identification. Sci. Rep..

[16] Li R., Shen X., Chen H., Peng D., Wu R., Sun H. (2021). Developmental validation of the MGIEasy Signature Identification Library Prep Kit, an all-in-one multiplex system for forensic applications. Int. J. Legal Med..

[17] Chen Y., Chen Y., Shi C., Huang Z., Zhang Y., Li S., Li Y., Ye J., Yu C., Li Z. (2018). SOAPnuke: A MapReduce acceleration-supported software for integrated quality control and preprocessing of high-throughput sequencing data. Gigascience.

[18] Li H. (2018). Minimap2: Pairwise alignment for nucleotide sequences. Bioinformatics.

[19] Khanna A., Larson D., Srivatsan S., Mosior M., Abbott T., Kiwala S., Ley T., Duncavage E., Walter M., Walker J. (2022). Bam-readcount—Rapid generation of basepair-resolution sequence metrics. J. Open Source Softw..

[20] Woerner A.E., King J.L., Budowle B. (2017). Fast STR allele identification with STRait Razor 3.0. Forensic Sci. Int. Genet..

[21] Lang Y., Guo F., Niu Q. (2019). StatsX v2.0: The interactive graphical software for population statistics on X-STR. Int. J. Legal. Med..

[22] Gouy A., Zieger M. (2017). STRAF—A convenient online tool for STR data evaluation in forensic genetics. Forensic Sci. Int. Genet..

[23] Wang Y., Liu C., Zhang C.C., Li R., Li Y., Ou X.L., Sun H.Y. (2015). Analysis of 17 Y-STR loci haplotype and Y-chromosome haplogroup distribution in five Chinese ethnic groups. Electrophoresis.

[24] Paradis E. (2010). Pegas: An R package for population genetics with an integrated-modular approach. Bioinformatics.

[25] Bherer C., Campbell C.L., Auton A. (2017). Refined genetic maps reveal sexual dimorphism in human meiotic recombination at multiple scales. Nat. Commun..

[26] Kosambi D.D. (2016). The Estimation of Map Distances from Recombination Values.

[27] Ginestet C. (2011). ggplot2: Elegant Graphics for Data Analysis. J. R. Stat. Soc. Ser. A Stat. Soc..

[28] Lan Q., Zhao C., Chen C., Xu H., Fang Y., Yao H., Zhu B. (2022). Forensic Feature Exploration and Comprehensive Genetic Insights Into Yugu Ethnic Minority and Northern Han Population via a Novel NGS-Based Marker Set. Front. Genet..

[29] Aceves M.E.G., Cortés G.M., Villalobos H.R. (2017). Results obtained in five years in a paternity testing laboratory in Mexico. Forensic Sci. Int. Genet. Suppl. Ser..

[30] Amorim A., Pereira L. (2005). Pros and cons in the use of SNPs in forensic kinship investigation: A comparative analysis with STRs. Forensic Sci. Int..

[31] Kayser M. (2017). Forensic use of Y-chromosome DNA: A general overview. Hum. Genet..

[32] Parsons T.J., Huel R.M.L., Bajunović Z., Rizvić A. (2019). Large scale DNA identification: The ICMP experience. Forensic Sci. Int. Genet..

[33] Jäger A.C., Alvarez M.L., Davis C.P., Guzmán E., Han Y., Way L., Walichiewicz P., Silva D., Pham N., Caves G. (2017). Developmental validation of the MiSeq FGx Forensic Genomics System for Targeted Next Generation Sequencing in Forensic DNA Casework and Database Laboratories. Forensic Sci. Int. Genet..

[34] Mo S.K., Ren Z.L., Yang Y.R., Liu Y.C., Zhang J.J., Wu H.J., Li Z., Bo X.C., Wang S.Q., Yan J.W. (2018). A 472-SNP panel for pairwise kinship testing of second-degree relatives. Forensic Sci. Int. Genet..

[35] Egeland T., Sheehan N. (2008). On identification problems requiring linked autosomal markers. Forensic Sci. Int. Genet..

[36] Epstein M.P., Duren W.L., Boehnke M. (2000). Improved inference of relationship for pairs of individuals. Am. J. Hum. Genet..

[37] Morimoto C., Manabe S., Fujimoto S., Hamano Y., Tamaki K. (2018). Discrimination of relationships with the same degree of kinship using chromosomal sharing patterns estimated from high-density SNPs. Forensic Sci. Int. Genet..

[38] Parker L.S., London A.J., Aronson J.D. (2013). Incidental findings in the use of DNA to identify human remains: An ethical assessment. Forensic Sci. Int. Genet..

[39] (2021). Technical Specification for Identification of Biological Full Sibling Relationship.

[40] Cui W., Chen M., Yang Y., Cai M., Lan Q., Xie T., Zhu B. (2023). Applications of 1993 single nucleotide polymorphism loci in forensic pairwise kinship identifications and inferences. Forensic Sci. Int. Genet..

[41] Galván-Femenía I., Barceló-Vidal C., Sumoy L., Moreno V., de Cid R., Graffelman J. (2021). A likelihood ratio approach for identifying three-quarter siblings in genetic databases. Heredity.

[42] Kling D., Welander J., Tillmar A., Skare Ø., Egeland T., Holmlund G. (2012). DNA microarray as a tool in establishing genetic relatedness—Current status and future prospects. Forensic Sci. Int. Genet..

[43] Li H., Glusman G., Hu H., Shankaracharya, Caballero J., Hubley R., Witherspoon D., Guthery S.L., Mauldin D.E., Jorde L.B. (2014). Relationship Estimation from Whole-Genome Sequence Data. PLoS Genet..

