# Supplementary figures and images for: Inferring the Degree of Relatedness and Kinship Types Using an All-in-One Marker Set
Source: Genes (Basel). 2025 Apr 15;16(4):455. doi: 10.3390/genes16040455 (PMC12026669; doi:10.3390/genes16040455)

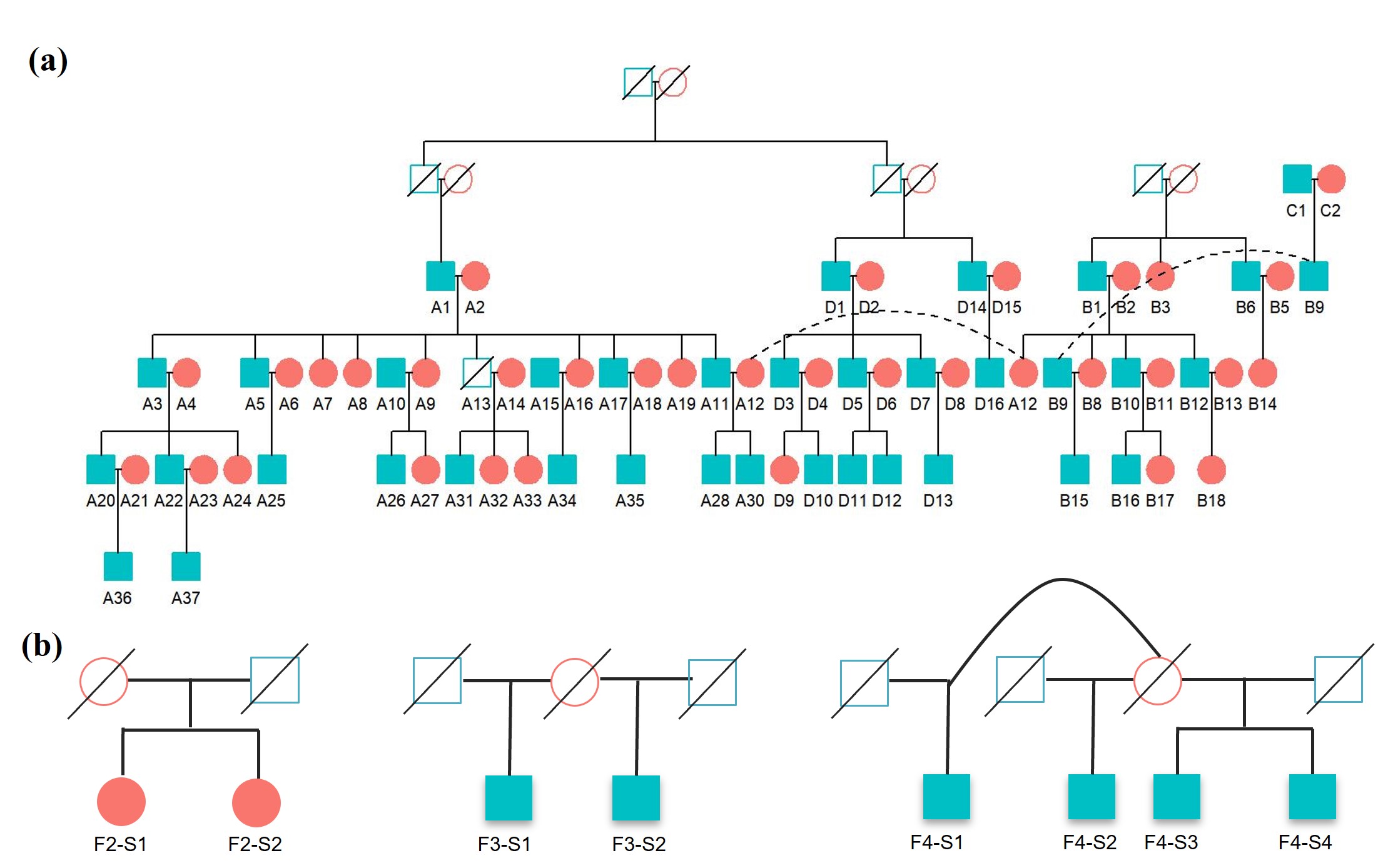

Supplement: Supplementary file 1 [file genes-16-00455-s001.zip › genes-3563755-supplementary/Figure S1. pedigrees of studied family.jpg]

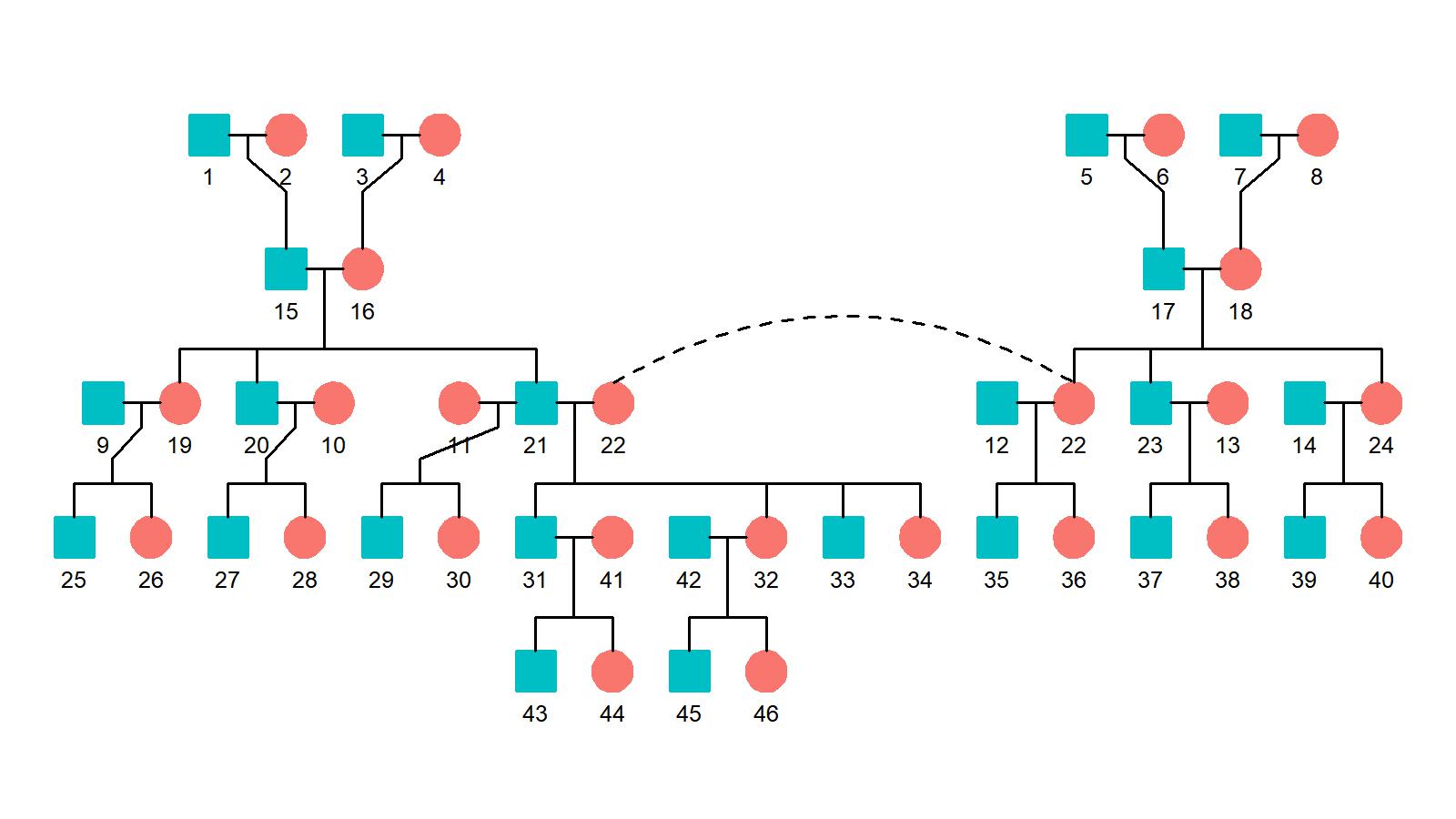

Supplement: Supplementary file 1 [file genes-16-00455-s001.zip › genes-3563755-supplementary/Figure S2. simulated pedigrees.jpeg]

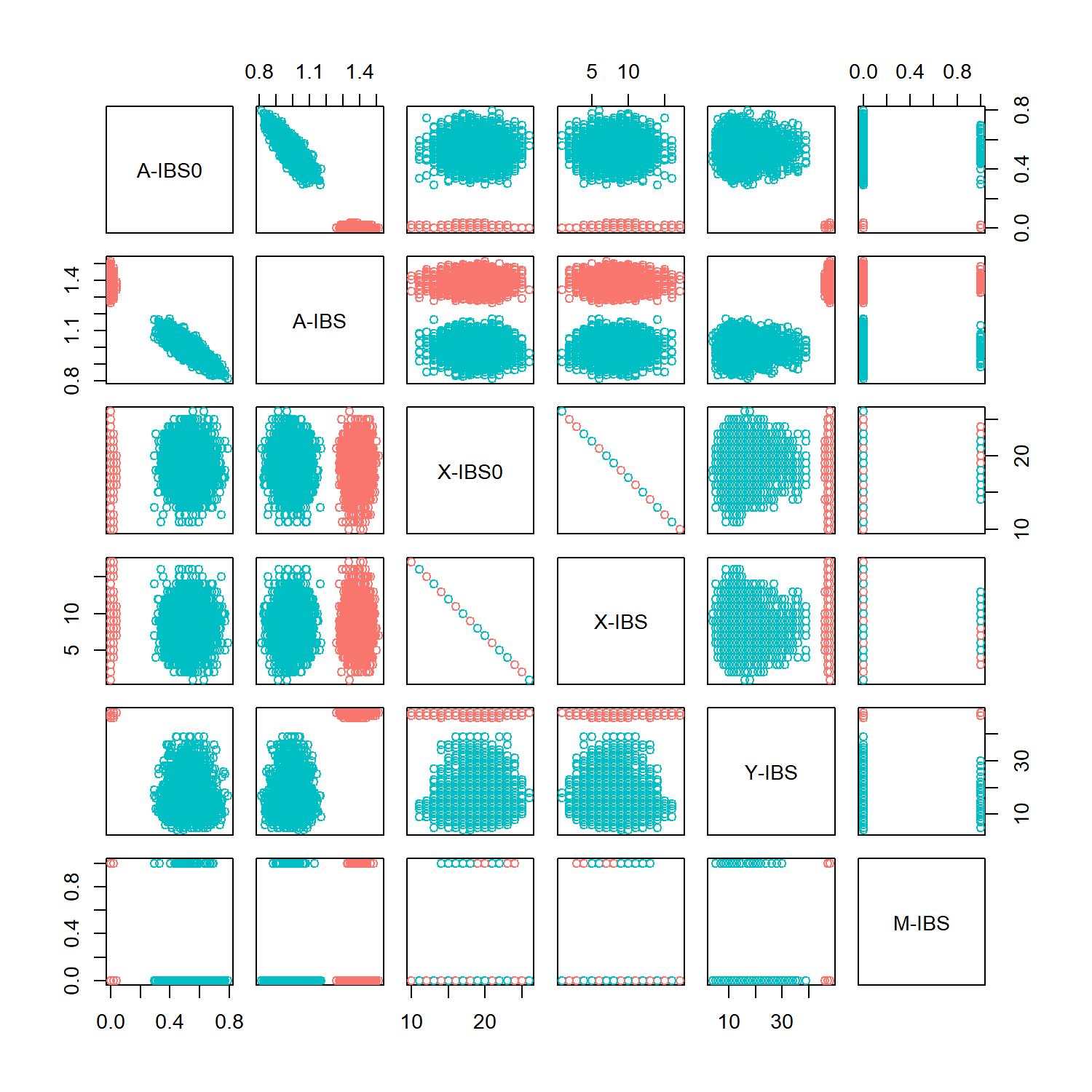

Supplement: Supplementary file 1 [file genes-16-00455-s001.zip › genes-3563755-supplementary/Figure S3. pairs of IBS.jpeg]

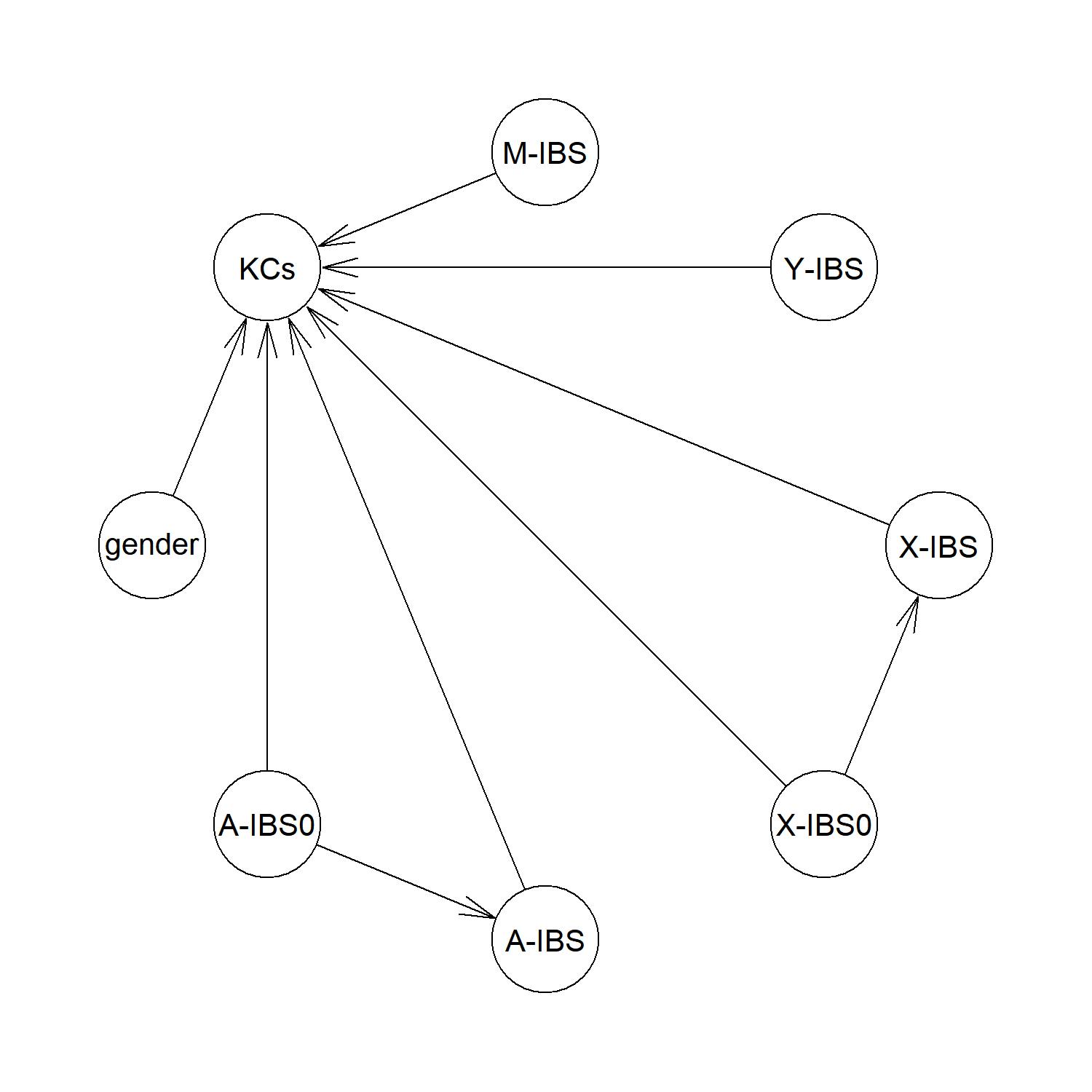

Supplement: Supplementary file 1 [file genes-16-00455-s001.zip › genes-3563755-supplementary/Figure S4. an example of BN .jpeg]

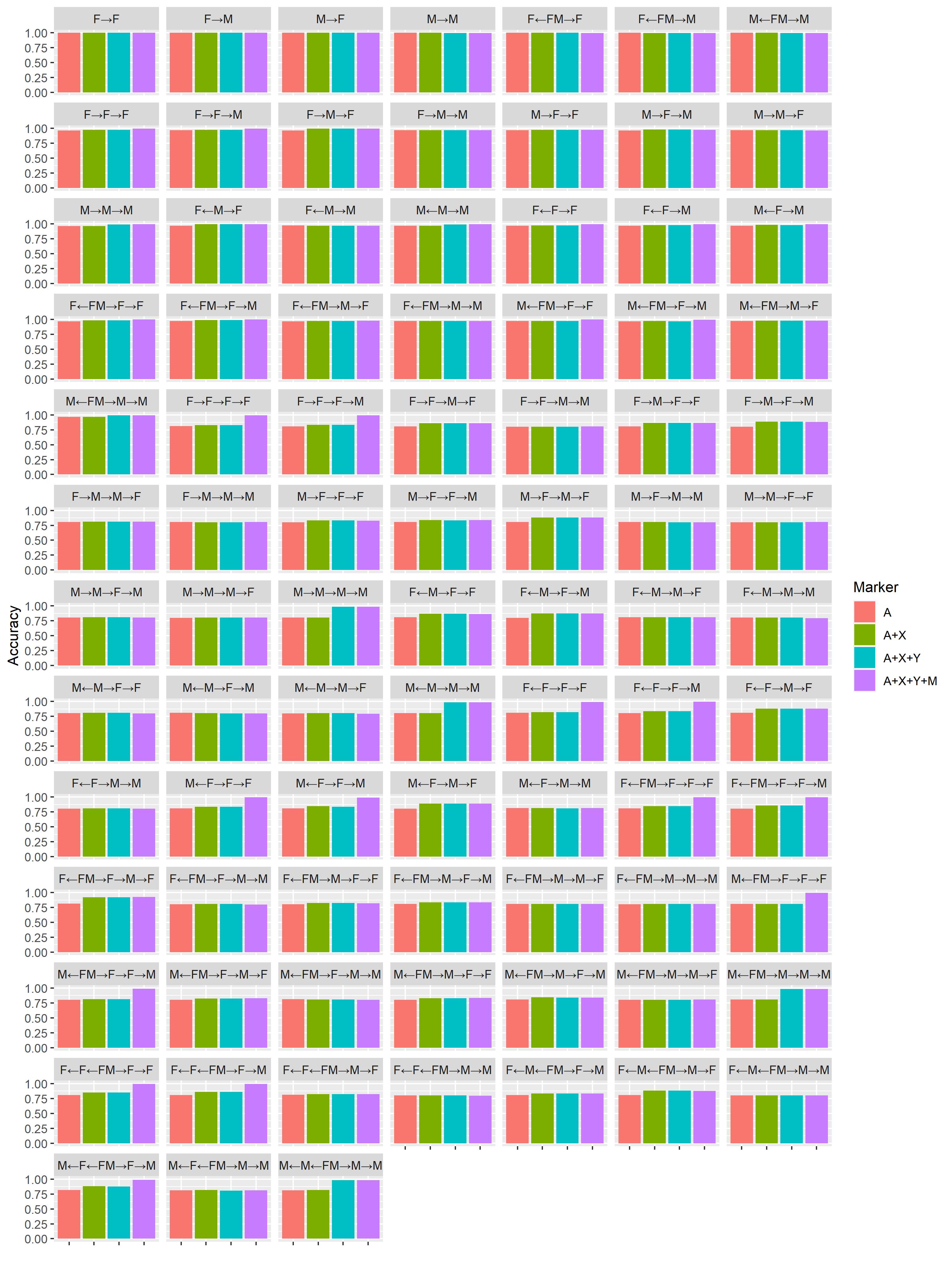

Supplement: Supplementary file 1 [file genes-16-00455-s001.zip › genes-3563755-supplementary/Figure S5. accuracy with UN.jpeg]

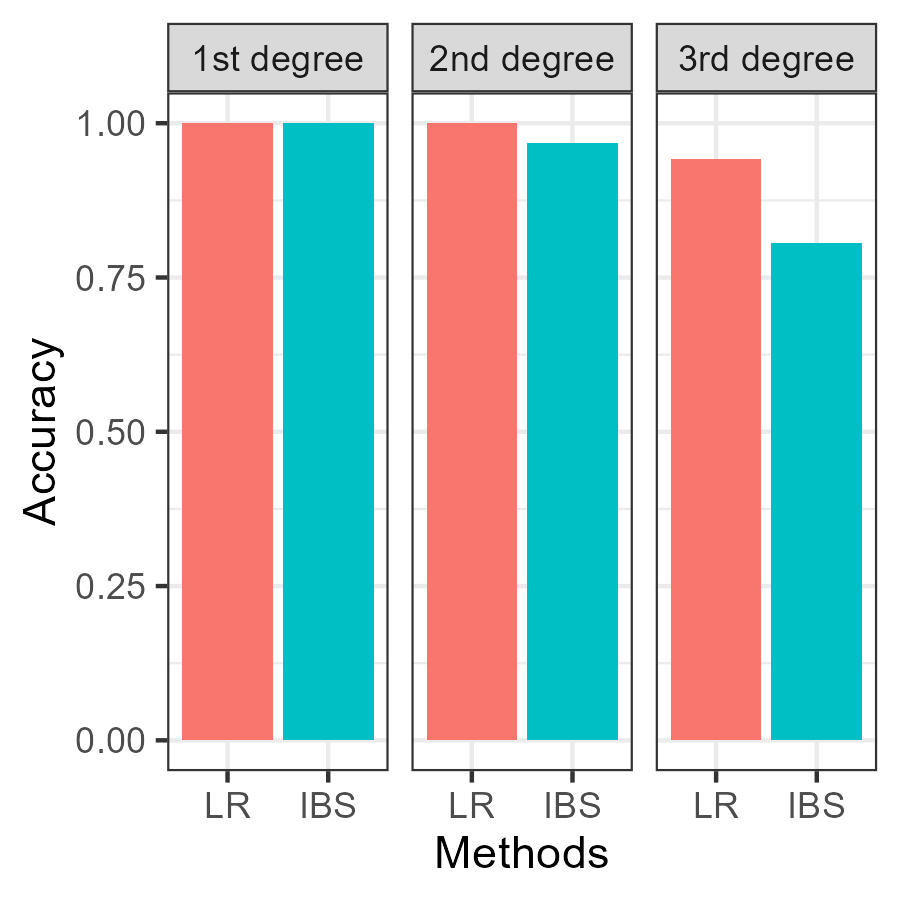

Supplement: Supplementary file 1 [file genes-16-00455-s001.zip › genes-3563755-supplementary/Figure S6. lr and ibs.jpeg]
